# Supplementary material for: Autotoxicity of Endogenous Organic Acid Stress in Two Ganoderma lucidum Cultivars
Source: Molecules. 2022 Oct 9;27(19):6734. doi: 10.3390/molecules27196734 (PMC9570943; doi:10.3390/molecules27196734)
Supplement: Supplementary file 1 [file molecules-27-06734-s001.zip › molecules-1901994-supplementary.pdf]

Table S1 Organic acids and derivatives in germ substrates of two cultivars by positive MS^E continum

No.	Differential compounds	Retention time(min)	Formula	Content change
1	beta-Alanyl-L-arginine	0.92	C9H19N5O3	DOWN
2	L-Citrulline	0.93	C6H13N3O3	DOWN
3	D-Gulonic acid	1.02	C6H12O7	DOWN
4	N-Acetylneuraminic acid	1.04	C11H19NO9	DOWN
5	pantethine	1.05	C22H42N4O8S2	DOWN
6	4-Methyleneglutamic acid	1.08	C6H9NO4	DOWN
7	N-Heptanoylglycine	1.13	C9H17NO3	UP
8	N-Acetyl-4-O-acetylneuraminic acid	1.13	C13H21NO10	DOWN
9	L-dopaquinone	1.14	C9H9NO4	UP
10	Isonicotinic acid	1.15	C6H5NO2	UP
11	(2S)-3-Methyl-2-({[(3S,4S,5R)-2,3,4-trihydroxy-5-(hydroxymethyl)tetrahydro-2-furanyl]methyl}amino)butanoic acid	1.19	C11H21NO7	DOWN
12	N-Acetylmuramic acid	1.25	C11H19NO8	DOWN
13	pantothenic acid	1.50	C9H17NO5	UP
14	4-Guanidinobutanoic acid	1.57	C5H11N3O2	UP
15	linatine	2.06	C10H17N3O5	UP
16	2-Acetamidohexanedioic acid	2.25	C8H13NO5	UP
17	Glutarylglycine	2.36	C7H11NO5	UP
18	3-Methylhippuric Acid	2.49	C10H11NO3	DOWN
19	5,5'-[Disulfanediylbis({3-[(carboxymethyl)amino]-3-oxo-1,2-propanediyl}imino)]bis(2-amino-5-oxopentanoic acid)	2.50	C20H32N6O12S2	DOWN

20	4-Undecylbenzenesulfonic acid	2.52	C17H28O3S	DOWN
21	(E)-p-coumaric acid	2.56	C9H8O3	DOWN
22	(2S)-6-Amino-2-[(E)-(hydroxymethylene)amino]hexanimidic acid	2.63	C7H15N3O2	UP
23	2-Amino-6-[(E)-(5-amino-5-carboxy-2-hydroxypentylidene)amino]-5-hydroxyhexanoic acid	2.65	C12H23N3O6	DOWN
24	3,3',3'',3''',3''''-[8,13-Bis(carboxymethyl)-18-methyl-2,3,7,12,17-porphyrinpentayl]pentapropanoic acid	2.74	C40H40N4O14	DOWN
25	1,4'-Bipiperidine-1'-carboxylic acid	2.87	C11H20N2O2	DOWN
26	1-{[(3S,4S,5R)-2,3,4-Trihydroxy-5-(hydroxymethyl)tetrahydro-2-furanyl]methyl}-2-pyrrolidinecarboxylic acid	2.90	C11H19NO7	DOWN
27	Pipecolic acid	2.94	C6H11NO2	UP
28	(6S)-2-Amino-6-[(3-carboxypropanoyl)amino]heptanedioic acid	2.95	C11H18N2O7	UP
29	(2E)-3-(6-Hydroxy-2,3,4-trimethoxyphenyl)acrylic acid	2.98	C12H14O6	UP
30	N6,N6,N6-Trimethyl-L-lysine	3.05	C9H20N2O2	DOWN
31	Prolylglycine	3.09	C7H12N2O3	DOWN
32	(2E)-3-(7-Hydroxy-6-methoxy-1,3-benzodioxol-5-yl)acrylic acid	3.14	C11H10O6	UP
33	4-[(3-Acetamidopropyl)amino]butanoic acid	3.17	C9H18N2O3	DOWN
34	Methionyl-Proline	3.18	C10H18N2O3S	DOWN
35	(2E)-2-Hexenedioic acid	3.19	C6H8O4	DOWN
36	Coprine	3.22	C8H14N2O4	UP
37	6-Acetamido-2-oxohexanoic acid	3.25	C8H13NO4	UP
38	2-Hydroxy-3-(3,4,5-trihydroxyphenyl)propanoic acid	3.27	C9H10O6	UP
39	2-methoxy-17beta-estradiol 3-glucosiduronic acid	3.29	C25H34O9	DOWN
40	L-Phenylalanine	3.29	C9H11NO2	UP
41	2-methylcitric acid	3.32	C7H10O7	UP
42	Ascorbalamic acid	3.34	C9H13NO8	DOWN
43	O-Acetyl-L-serine	3.36	C5H9NO4	DOWN

44	(6alpha,8xi,11beta,14xi,16alpha,17alpha)-6,9-Difluoro-11,17-dihydroxy-16-methyl-3-oxoandrosta-1,4-diene-17-carboxylic acid	3.38	C21H26F2O5	DOWN
45	(2S)-5-[(2R)-6-Hydroxy-2,7,8-trimethyl-3,4-dihydro-2H-chromen-2-yl]-2-methylpentanoic acid	3.43	C18H26O4	DOWN
46	L-alpha-Amino-1H-pyrrole-1-hexanoic acid	3.48	C10H16N2O2	DOWN
47	Hydroxyprolyl-Lysine	3.49	C11H21N3O4	DOWN
48	N-4-hydroxyphenylacetylglutamic acid	3.49	C13H15NO6	UP
49	L-2-succinylamino-6-oxoheptanedioic acid	3.58	C11H15NO8	UP
50	4-Aminobenzoic acid	3.62	C7H7NO2	UP
51	3-{2-[(2E)-3,7-Dimethyl-2,6-octadien-1-yl]-3,4,5-trihydroxyphenyl}propanoic acid	3.67	C19H26O5	DOWN
52	Triethyl citrate	3.76	C12H20O7	UP
53	3-(4-Hydroxy-3,5-dimethoxyphenyl)-2-oxiranecarboxylic acid	3.80	C11H12O6	UP
54	2-Acetoxy-3-[3-(4-hydroxy-phenyl)-acryloyloxy]-succinic acid	3.84	C15H14O9	UP
56	1-O-[(2E)-3-(3,4-Dihydroxyphenyl)-2-propenoyl]-beta-D-glucopyranuronic acid	3.84	C15H16O10	UP
57	5-hydroxy-2-oxo-4-ureido-2,5-dihydro-1*H*-imidazole-5-carboxylic acid	3.88	C5H6N4O5	UP
58	Glycylproline	3.91	C7H12N2O3	DOWN
59	Glutamylphenylalanine	3.93	C14H18N2O5	DOWN
60	(4Z,7Z,10S,11E)-10-Hydroperoxy-12-{(1R,5S,6R)-6-[(2Z)-2-penten-1-yl]-2,3-dioxabicyclo[2.2.1]hept-5-yl}-4,7,11-dodecatrienoic acid	3.96	C22H32O6	DOWN
61	2-[(3Z)-4,8-Dimethyl-3,7-nonadien-1-yl]-8-hydroxy-3-methylene-6-chromanecarboxylic acid	3.97	C22H28O4	DOWN
62	Vanillic acid-4-O-glucuronide	4.01	C15H18O9	UP
63	3-Hydroxy-3-carboxymethyl-adipic acid	4.07	C8H12O7	UP
64	3-[(1E)-3-Hydroxy-1-propen-1-yl]phenyl hexopyranosiduronic acid	4.12	C15H18O8	UP
65	4-Hydroxy-5-(3,4,5-trihydroxyphenyl)pentanoic acid	4.16	C11H14O6	UP
66	(2E)-3-(3,5-Dihydroxy-4-methoxyphenyl)acrylic acid	4.17	C10H10O5	UP
67	N-{[5-Amino-1-(5-O-phosphonopentofuranosyl)-1H-imidazol-4-yl]carbonyl}aspartic acid	4.17	C13H19N4O12P	UP
68	Fertaric acid	4.19	C14H14O9	UP

69	4-(2-Aminophenyl)-2,4-dioxobutanoic acid	4.30	C10H9NO4	UP
70	4-hydroxy-4-(indol-3-ylmethyl)glutamic acid	4.31	C14H16N2O5	UP
71	piscidic acid	4.35	C11H12O7	UP
72	4-Hydroxy-3-nitrophenylacetic acid	4.37	C8H7NO5	UP
73	(4-Methoxy-1-benzofuran-5-yl)(oxo)acetic acid	4.42	C11H8O5	UP
74	{[(2E)-3-(3,4-Dihydroxyphenyl)-2-propenoyl]oxy}malonic acid	4.43	C12H10O8	UP
75	3-(2,4-Dihydroxyphenyl)-2-oxopropanoic acid	4.45	C9H8O5	UP
76	2-(2-Carboxyethyl)-5-hydroxyphenyl hexopyranosiduronic acid	4.47	C15H18O10	UP
77	3,4,5-Trimethoxycinnamic acid	4.52	C12H14O5	UP
78	DL-gamma-carboxyglutamic acid	4.59	C6H9NO6	UP
79	4-[(E)-2-Carboxyvinyl]-2-methoxyphenyl beta-D-glucopyranosiduronic acid	4.61	C16H18O10	UP
80	3,4-Dihydroxy-5-[(2E,6Z)-3,7,11-trimethyl-2,6,10-dodecatrien-1-yl]benzoic acid	4.68	C22H30O4	DOWN
81	2-(3,4-dihydroxybenzoyloxy)-4,6-dihydroxybenzoic acid	4.79	C14H10O8	UP
82	1-O-[(6-Hydroxy-1,3-benzodioxol-5-yl)carbonyl]hexopyranuronic acid	4.86	C14H14O11	UP
83	11-{3,4-Dimethyl-5-[(1E)-1-penten-1-yl]-2-furyl}undecanoic acid	4.91	C22H36O3	DOWN
84	3,3'-(2,2'-Dihydroxy-4,4'-biphenyldiyl)bis(2-aminopropanoic acid)	4.94	C18H20N2O6	DOWN
85	8-(2-Hydroxy-3-methyl-3-buten-1-yl)-2-oxo-2H-chromen-7-yl hexopyranosiduronic acid	5.04	C20H22O10	UP
86	Homocarnosine	5.05	C10H16N4O3	UP
87	(3E)-4-(1-Oxo-1H-isochromen-3-yl)-3-buten-1-yl hexopyranosiduronic acid	5.17	C19H20O9	UP
88	3-(3,4-Dihydroxyphenyl)-2-{[(2E)-3-(4-hydroxy-3-methoxyphenyl)-2-propenoyl]oxy}propanoic acid	5.19	C19H18O8	UP
89	(5E)-2,10-Diamino-5-{[(5-amino-5-carboxypentyl)amino]methyl}-5-undecenedioic acid	5.24	C18H34N4O6	DOWN
90	3-Hydroxy-2-(3-methyl-2-buten-1-yl)phenyl hexopyranosiduronic acid	5.25	C17H22O8	UP
91	3-Hydroxyicosanoic acid	5.30	C20H40O3	DOWN
92	(-)-Cryptochlorogenic acid	5.33	C16H18O9	UP
93	3-Hydroxy-5, 8-tetradecadiencarnitine	5.39	C21H37NO5	UP

94	Salvianolic acid D	5.41	C20H18O10	UP
95	3,5-Dihydroxy-2-[3-(4-hydroxyphenyl)propanoyl]phenyl beta-D-glucopyranosiduronic acid	5.41	C21H22O11	UP
96	3,4-Dihydroxy-2-(3-methyl-2-buten-1-yl)phenyl hexopyranosiduronic acid	5.54	C17H22O9	UP
97	1-O-[(2E)-3-(3-Hydroxy-4-methoxyphenyl)-2-propenoyl]-beta-D-glucopyranuronic acid	5.58	C16H18O10	UP
98	6-Hydroxy-7-oxo-3a,12c-dihydro-7H-furo[3',2':4,5]furo[2,3-c]xanthen-8-yl hexopyranosiduronic acid	5.59	C23H18O12	UP
99	4-Oxo-1-(5,6,7-trimethoxy-4-oxo-4H-chromen-2-yl)-2,5-cyclohexadien-1-yl hexopyranosiduronic acid	5.60	C24H24O13	UP
100	5,7-Dihydroxy-4-oxo-2-phenyl-3,4-dihydro-2H-chromen-6-yl hexopyranosiduronic acid	5.67	C21H20O11	UP
101	17-Hydroxylinolenic acid	5.69	C18H30O3	UP
102	3-C-Carboxy-2-deoxy-4-O-[(2E)-3-(4-hydroxyphenyl)-2-propenoyl]pentaric acid	5.76	C15H14O10	UP
103	3-[(1E)-4-Hydroxy-1-buten-1-yl]-1-oxo-1H-isochromen-5-yl hexopyranosiduronic acid	5.78	C19H20O10	UP
104	2-Amino-3-{[({2-carboxy-2-[(Z)-(1-hydroxyethylidene)amino]ethyl}sulfanyl)methyl]sulfanyl}propanoic acid	5.79	C9H16N2O5S2	UP
105	6-Hydroxy-7-oxo-1,2,3a,12c-tetrahydro-7H-furo[3',2':4,5]furo[2,3-c]xanthen-8-yl hexopyranosiduronic acid	5.81	C23H20O12	UP
106	2-Benzyl-2,4,6-trihydroxy-3-oxo-2,3-dihydro-1-benzofuran-5-yl hexopyranosiduronic acid	5.82	C21H20O12	UP
107	4-[(2R)-2-(Aminomethyl)-2-(hydroxymethyl)-5-oxo-1-pyrrolidinyl]-3-(3-pentanylamino)benzoic acid	5.85	C18H27N3O4	DOWN
108	Traumatic Acid	5.85	C12H20O4	DOWN
109	2-Methoxy-5-[(2R,3R)-3,5,7-trihydroxy-3,4-dihydro-2H-chromen-2-yl]phenyl beta-D-glucopyranosiduronic acid	5.87	C22H24O12	UP
110	2-Hydroxy-3-[4-hydroxy-3-methoxy-5-(3-methyl-2-buten-1-yl)phenyl]propanoic acid	5.89	C15H20O5	UP
111	5-Hydroxydecanedioic acid	5.91	C10H18O5	DOWN
112	5-Formiminotetrahydrofolic acid	6.11	C20H24N8O6	UP
113	2-(Phenoxycarbonyl)phenyl hexopyranosiduronic acid	6.13	C19H18O9	UP

114	(2E)-3-(3-Hydroxy-2,4-dimethoxyphenyl)acrylic acid	6.14	C11H12O5	UP
115	3-[(1E)-1-Buten-1-yl]-1-oxo-1H-isochromen-5-yl hexopyranosiduronic acid	6.16	C19H20O9	UP
116	(6S)-2,6-Anhydro-6-[(1S)-2-isopropyl-5-methylcyclohexyl]-L-gulonic acid	6.24	C16H28O6	UP
117	7-[(1R,5S)-6-Amino-3-azabicyclo[3.1.0]hex-3-yl]-1-(2,4-difluorophenyl)-6-fluoro-4-oxo-1,4-dihydro-1,8-naphthyridine-3-carboxylic acid	6.28	C20H15F3N4O3	UP
118	2-Amino-6-({[2,3,4-trihydroxy-5-(hydroxymethyl)tetrahydro-2-furanyl]methyl}amino)hexanoic acid	6.29	C12H24N2O7	UP
119	3,6,7-Trihydroxy-2-(4-hydroxy-3-methoxyphenyl)-8aH-chromen-5-yl hexopyranosiduronic acid	6.33	C22H22O13	UP
120	6-Hydroxypentadecanedioic acid	6.35	C15H28O5	UP
121	1-(2-Carboxyethyl)-2,3,4,9-tetrahydro-1*H*-beta-carboline-3-carboxylic acid	6.37	C15H16N2O4	UP
122	5-Hydroxy-2-(3-hydroxy-4-methoxyphenyl)-4-oxo-3,4-dihydro-2H-chromen-7-yl beta-D-glucopyranosiduronic acid	6.51	C22H22O12	UP
123	4-(5-Hydroxy-2-methyl-2-azabicyclo[2.2.2]oct-5-yl)-3-methylbutanoic acid	6.52	C13H23NO3	DOWN
124	7-Hydroxy-1,4a-dimethyl-8-methylenegibbane-1,10-dicarboxylic acid	6.54	C20H28O5	DOWN
125	Myristoylglycine	6.57	C16H31NO3	DOWN
126	7-Hydroxyhexadecanedioic acid	6.57	C16H30O5	UP
127	4-(5,7-Dihydroxy-3,6-dimethoxy-4-oxo-4H-chromen-2-yl)-2-methoxyphenyl beta-D-glucopyranosiduronic acid	6.59	C24H24O14	UP
128	5-Hydroxy-4-(6-hydroxy-3-oxo-2,3-dihydro-1-benzofuran-2-yl)-3-methoxy-2-(3-methyl-2-buten-1-yl)phenyl hexopyranosiduronic acid	6.64	C26H28O12	UP
129	4a-Formyl-3,7-dihydroxy-1-methyl-8-methylenegibbane-1,10-dicarboxylic acid	6.71	C20H26O7	UP
130	3,7,15-Trihydroxy-11,23-dioxolanost-8-en-26-oic acid	6.80	C30H46O7	UP
131	7-(3-Methyl-5-propyl-2-furyl)heptanoic acid	6.82	C15H24O3	DOWN
132	(9alpha,11alpha,13E,15S)-5,6,9,11,15-Pentahydroxyprost-13-en-1-oic acid	6.86	C20H36O7	UP
133	(24E)-3,7,12,23-Tetrahydroxy-11,15-dioxolanosta-8,24-dien-26-oic acid	6.90	C30H44O8	UP
134	4-(5,6-Dihydroxy-3-oxo-2,3-dihydro-1-benzofuran-2-yl)-5-hydroxy-3-methoxy-2-(3-methyl-2-bute	6.95	C26H28O13	UP

	n-1-yl)phenyl hexopyranosiduronic acid			
135	4-(5,7-Dihydroxy-4-oxo-3,4-dihydro-2H-chromen-2-yl)phenyl beta-D-erythro-hexopyranosiduronic acid	6.95	C21H20O11	UP
136	2-(6-Carboxy-5-{3-[3-(2,4-dihydroxyphenyl)propanoyl]-2,6-dihydroxyphenyl}-3-methyl-3-cyclohexen-1-yl)-5-hydroxyphenyl hexopyranosiduronic acid	6.96	C35H36O15	DOWN
137	4a-Formyl-7,9-dihydroxy-1-methyl-8-methylenegibbane-1,10-dicarboxylic acid	7.01	C20H26O7	UP
138	3,5-Dihydroxy-4-(3-methyl-2-buten-1-yl)-2-[3-(2,4,5-trihydroxyphenyl)propanoyl]phenyl hexopyranosiduronic acid	7.10	C26H30O13	UP
139	Hexadecanedioic acid mono-L-carnitine ester	7.10	C23H43NO6	DOWN
140	7,7'-Dihydroxy-5'-methoxy-2',2'-dimethyl-4-oxo-3',4'-dihydro-2'H,4H-3,6'-bichromen-5-yl hexopyranosiduronic acid	7.12	C27H28O13	UP
141	4-[2-(2,4-Dihydroxyphenyl)-2-oxoethyl]-5-hydroxy-2-[(2E)-4-hydroxy-3-methyl-2-buten-1-yl]-3-methoxyphenyl hexopyranosiduronic acid	7.25	C26H30O13	UP
142	1-O-[(2S)-2-(6-Methoxy-2-naphthyl)propanoyl]-D-glucopyranuronic acid	7.30	C20H22O9	UP
143	2-Deoxy-4-O-[(2E)-3-(4-hydroxyphenyl)-2-propenoyl]pentaric acid	7.31	C14H14O8	UP
144	2-(3-Hydroxybenzyl)succinic acid	7.32	C11H12O5	UP
145	(2R)-1-[(2S,4R,5Z)-4-Benzyl-2,5-dihydroxy-5-{[(1R,2S)-2-hydroxy-2,3-dihydro-1H-inden-1-yl]imino}pentyl]-N-(2-methyl-2-propanyl)-2-piperazinecarboximidic acid	7.37	C30H42N4O4	UP
146	2,6-Anhydro-6-[1-(1,3-benzodioxol-5-yl)-3-(4-methoxy-1-benzofuran-5-yl)-1,3-dioxo-2-propanyl] hexonic acid	7.41	C25H22O12	UP
147	8,10-Dihydroxy-11-(3-methyl-2-buten-1-yl)-6-(2-methyl-1-propen-1-yl)-7-oxo-6H,7H-chromeno[4,3-b]chromen-3-yl hexopyranosiduronic acid	7.45	C31H32O12	DOWN
148	Mevalonic acid-5P	7.49	C6H13O7P	UP
149	2-[2-(2,4-Dihydroxyphenyl)-2-oxoethyl]-5-hydroxy-3-methoxy-4-(3-methyl-2-buten-1-yl)phenyl hexopyranosiduronic acid	7.58	C26H30O12	UP
150	2,6-Anhydro-6-[1-(3,4-dihydroxyphenyl)-3-(4-methoxy-1-benzofuran-5-yl)-1,3-dioxo-2-propanyl]	7.71	C24H22O12	UP

	hexonic acid			
151	5-Hydroxy-2,2-dimethyl-6-oxo-8-phenyl-1a,9c-dihydro-2H,6H-oxireno[c]pyrano[2,3-f]chromen-7-yl hexopyranosiduronic acid	7.76	C26H24O12	UP
152	2-[(2E)-3,7-Dimethyl-2,6-octadien-1-yl]-3,4,6-trihydroxybenzoic acid	7.78	C17H22O5	UP
153	Elaidolinolenic acid	7.87	C18H30O2	DOWN
154	(2E,6E,10E)-13-[(2R)-6-Hydroxy-2,7,8-trimethyl-3,4-dihydro-2H-chromen-2-yl]-2,6,10-trimethyl-2,6,10-tridecatrienoic acid	7.90	C28H40O4	UP
155	(8xi,9xi,14beta,17xi)-7-Hydroxy-3-oxocholan-24-oic acid	7.99	C24H38O4	UP
156	3-Hydroxy-5-({2-hydroxy-5-oxo-6-[(5,5,8a-trimethyl-2-methylenedecahydro-1-naphthalenyl)methyl]-7-oxabicyclo[4.1.0]hept-3-en-3-yl}methoxy)-3-methyl-5-oxopentanoic acid	8.14	C28H40O8	UP
157	3-Hydroxytetradecanedioic acid	8.53	C14H26O5	UP
158	b-D-Glucopyranosiduronic acid, (3a,5b)-24-[(carboxymethyl)amino]-24-oxocholan-3-yl	8.73	C26H43NO4	DOWN
159	2-Hydroxytetracosanoic acid	8.76	C24H48O3	UP
160	8-oxo-9,11-octadecadiynoic acid	8.95	C18H26O3	DOWN
161	(6E,8S,10Z)-8-Hydroxy-3-oxo-6,10-hexadecadienoic acid	8.97	C16H26O4	DOWN
162	[(2R,2'R,3R,4'R,4a'S,5S,5'R,6'R,8a'R)-5-(3-Furyl)-2,4'-dihydroxy-2',4a',5',8a'-tetrakis(hydroxymethyl)-6'-{[(2E)-2-methyl-2-butenoyl]oxy}decahydro-2'H-spiro[furan-3,1'-naphthalen]-5'-yl]acetic acid	9.01	C28H40O12	UP
163	(10E,12E,14E)-9-Hydroxy-16-oxo-10,12,14-octadecatrienoic acid	9.07	C18H28O4	DOWN
164	1,3,16,20,24-Pentahydroxy-24-(hydroxymethyl)-9,19-cyclolanostan-28-oic acid	9.10	C31H52O8	DOWN
165	16-Acetoxy-17-methoxy-17-oxokauran-18-oic acid	9.17	C23H34O6	DOWN
166	3,7,15-Trihydroxy-11,23-dioxolanost-8-en-26-oic acid	9.48	C30H46O7	UP
167	Prostan-1-oicacid, 9,11,15-trihydroxy-, (9a,11a,15S)-	9.49	C20H38O5	DOWN
168	Capsiamide	9.61	C17H35NO	DOWN
169	3-Hydroxyhexadecadienoylcarnitine	9.68	C23H41NO5	DOWN
170	9-Hexadecenoylcarnitine	10.18	C23H43NO4	DOWN
171	Phloionolic acid	10.21	C18H36O5	DOWN

172	13-Hydroxy-9-methoxy-10-oxo-11-octadecenoic acid	10.45	C19H34O5	DOWN
173	(1Z,2Z)-N-(2-Hydroxy-2-{4-[(3-methyl-2-buten-1-yl)oxy]phenyl}ethyl)-3-phenyl-2-propenimidic acid	10.51	C22H25NO3	DOWN
174	(3beta,5xi,9xi)-3-Hydroxyoleana-11,13(18)-dien-30-oic acid	10.51	C30H46O3	UP
175	2-Hydroxy-8-(2-octyl-1-cyclopropen-1-yl)octanoic acid	10.54	C19H34O3	DOWN
176	3,7,15-Trihydroxy-11,23-dioxolanost-8-en-26-oic acid	10.61	C30H46O7	UP
177	Gamma-Linolenic acid	10.67	C18H30O2	DOWN
178	3,15-Dihydroxy-11,23-dioxolanost-8-en-26-oic acid	10.72	C30H46O6	UP
179	13-oxotrideca-9,11-dienoic acid	10.98	C13H20O3	DOWN
180	2,3-Dihydroxy-29-methoxy-29-oxoolean-12-en-28-oic acid	11.63	C31H48O6	UP
181	16-Hydroxy-10-oxohexadecanoic acid	11.70	C16H30O4	DOWN
182	2-Hydroxy-10-isopropenyl-3,3,5a,5b,12b-pentamethyloctadecahydrodicyclopenta[a,i]phenanthrene-1,7a(1H)-dicarboxylic acid	12.21	C30H46O5	UP
183	3-Acetoxy-24-hydroxylanosta-8,25-dien-21-oic acid	12.96	C32H50O5	DOWN
184	3-{[(2E)-3-(3,4-Dihydroxyphenyl)-2-propenoyl]oxy}-2-hydroxylup-20(29)-en-28-oic acid	13.08	C39H54O7	DOWN
185	5-Oxooctadecanoic acid	13.34	C18H34O3	DOWN
186	3-Hydroxy-2-{[(2E)-3-(4-hydroxy-3-methoxyphenyl)-2-propenoyl]oxy}urs-12-en-28-oic acid	13.39	C40H56O7	DOWN
187	16-Hydroxy-3-oxooleana-1,12-dien-28-oic acid	13.55	C30H44O4	UP
188	(6R,10S)-13-[(2R)-6-Hydroxy-2,7,8-trimethyl-3,4-dihydro-2H-chromen-2-yl]-2,6,10-trimethyltride canoic acid	13.65	C28H46O4	UP
189	11,12,13-Trinor-1,3,5-bisabolatrien-10-oic acid	13.66	C12H16O2	DOWN
190	Nisinic acid	13.72	C24H36O2	DOWN
191	(24E)-3-Acetoxy-15,22-dihydroxylanosta-7,9(11),24-trien-26-oic acid	13.82	C32H48O6	UP
192	3-Acetoxylanosta-8,24-dien-21-oic acid	13.95	C32H50O4	DOWN
193	2-[(8E,11E,14Z)-8,11,14-Heptadecatrien-1-yl]-6-hydroxybenzoic acid	15.27	C24H34O3	DOWN
194	(6E,8E,12E,14Z)-hexadeca-6,8,12,14-tetraen-10-ynoic acid	15.97	C16H20O2	UP

Table S2 Organic acids and derivatives in germ substrates of two cultivars by negative MS^E continum

No.	Differential compounds	Formula	Retention time(min)	Content change
1	5-(Hydroxymethyl)furoic acid	C6H6O4	2.33	UP
2	1,3,4-Trihydroxy-5-{[(2Z)-3-(4-hydroxy-3-methoxyphenyl)-2-propenoyl]oxy}cyclohexanecarboxylic acid	C17H20O9	2.94	UP
3	2-Deoxy-4-O-[(2E)-3-(4-hydroxyphenyl)-2-propenoyl]pentaric acid	C14H14O8	3.84	UP
4	3-C-Carboxy-2-deoxy-4-O-[(2E)-3-(4-hydroxyphenyl)-2-propenoyl]pentaric acid	C15H14O10	4.25	UP
5	2-(2-Carboxyethyl)-5-hydroxyphenyl hexopyranosiduronic acid	C15H18O10	4.45	UP
6	3-[(1-Carboxyvinyl)oxy]-4-hydroxybenzoic acid	C10H8O6	4.75	UP
7	5-hydroxy-2-oxo-4-ureido-2,5-dihydro-1*H*-imidazole-5-carboxylic acid	C5H6N4O5	5.29	UP
8	3,5-Dihydroxy-2-[3-(4-hydroxyphenyl)propanoyl]phenyl hexopyranosiduronic acid	C21H22O11	5.42	UP
9	{[(2E)-3-(3,4-Dihydroxyphenyl)-2-propenoyl]oxy}malonic acid	C12H10O8	5.87	UP
10	C19:1 (CIS-10) ACID	C19H36O2	7.99	DOWN

a

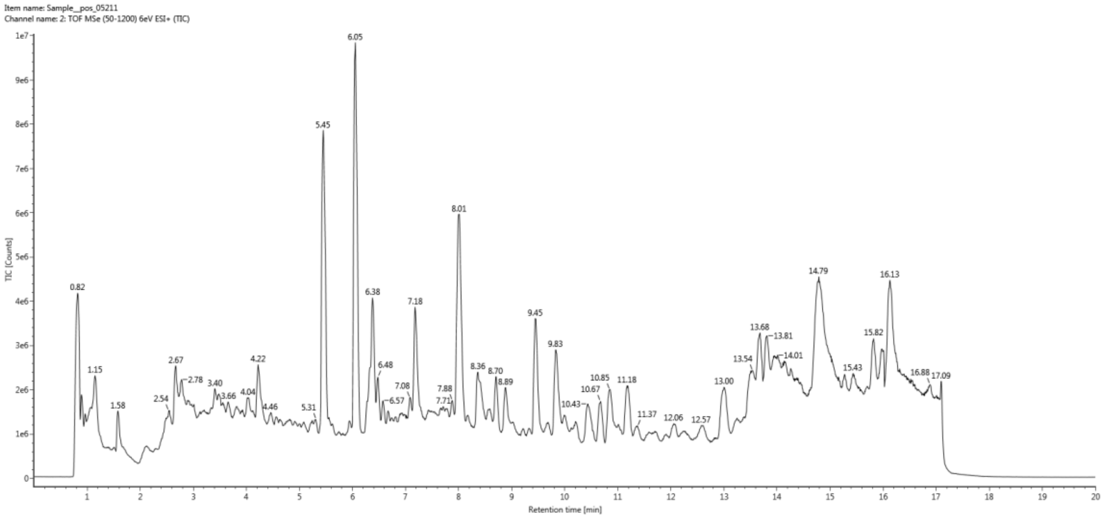
Item name: Sample__pos_05211
Channel name: 2: TOF MSe (50-1200) 6eV ESI+ (TIC)
TIC [Counts]
Retention time [min]
0.82
1.15
1.58
2.54
2.67
2.78
3.40
3.66
4.04
4.22
4.46
5.31
5.45
6.05
6.38
6.48
6.57
7.08
7.18
7.71
7.88
8.01
8.36
8.70
8.89
9.45
9.83
10.43
10.67
10.85
11.18
11.37
12.06
12.57
13.00
13.54
13.68
13.81
14.01
14.79
15.43
15.82
16.13
16.88
17.09

b

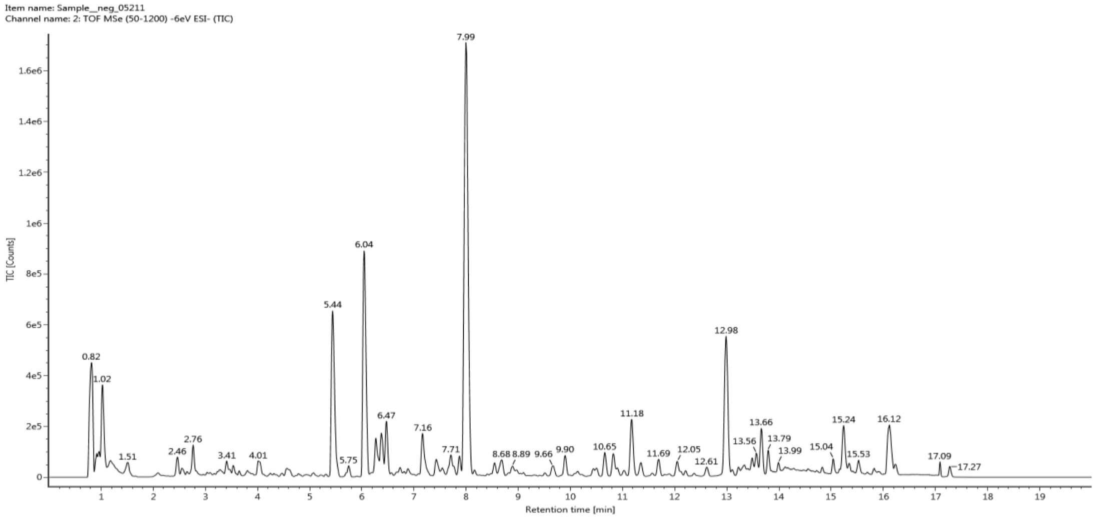
Item name: Sample__neg_05211
Channel name: 2: TOF MSe (50-1200) -6eV ESI- (TIC)
TIC [Counts]
Retention time [min]
0.82
1.02
1.51
2.46
2.76
3.41
4.01
5.44
5.75
6.04
6.47
7.16
7.71
7.99
8.68
8.89
9.66
9.90
10.65
11.18
11.69
12.05
12.61
12.98
13.56
13.66
13.79
13.99
15.04
15.24
15.53
16.12
17.09
17.27

c

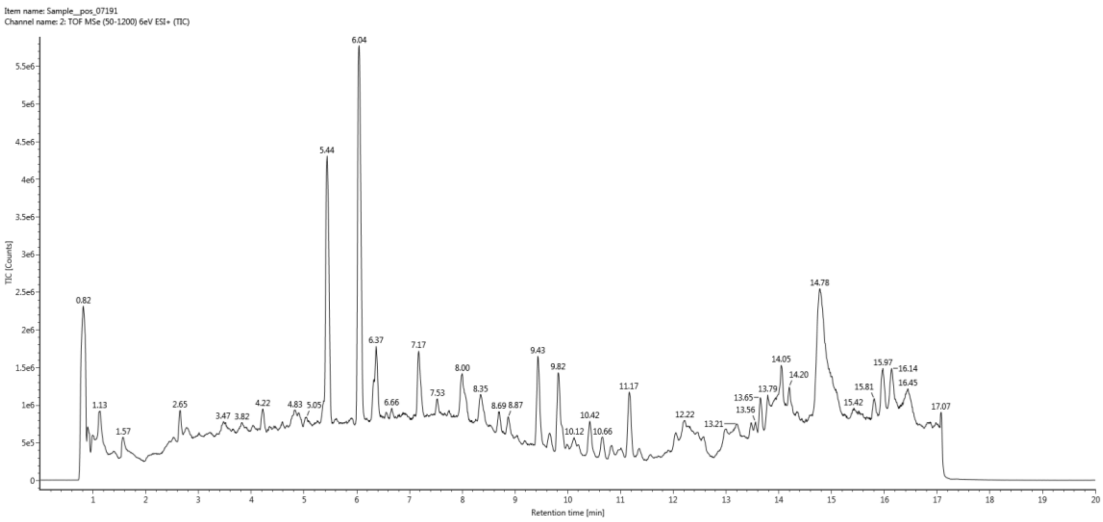
Item name: Sample__pos_07191
Channel name: 2: TOF MSe (50-1200) 6eV ESI+ (TIC)
TIC [Counts]
Retention time [min]
0.82
1.13
1.57
2.65
3.47
3.82
4.22
4.83
5.05
5.44
6.04
6.37
6.66
7.17
7.53
8.00
8.35
8.69
8.87
9.43
9.82
10.12
10.42
10.66
11.17
12.22
13.21
13.56
13.65
13.79
14.05
14.20
14.78
15.42
15.81
15.97
16.14
16.45
17.07

d

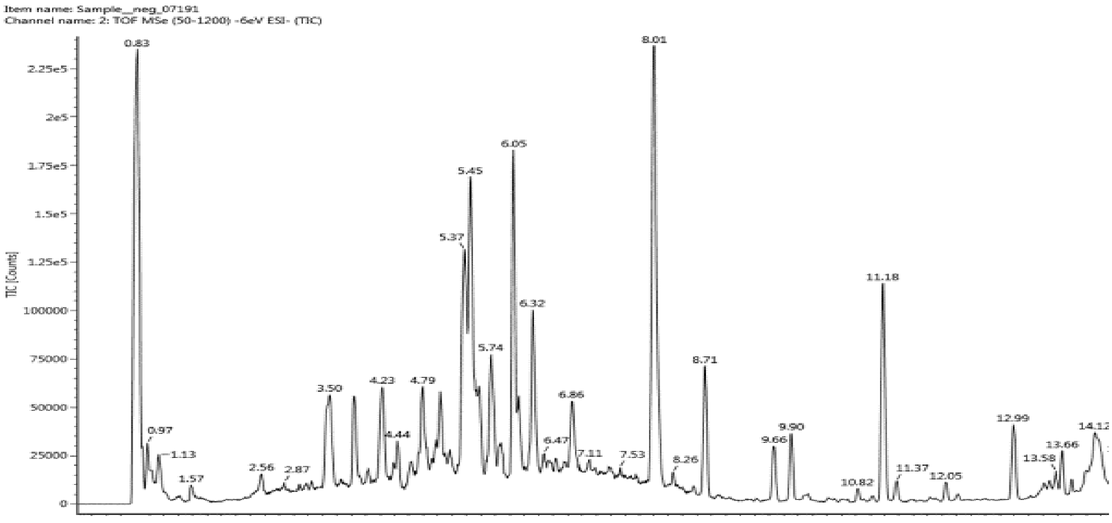
Item name: Sample__neg_07191
Channel name: 2: TOF MSe (50-1200) -6eV ESI- (TIC)
TIC [Counts]

e

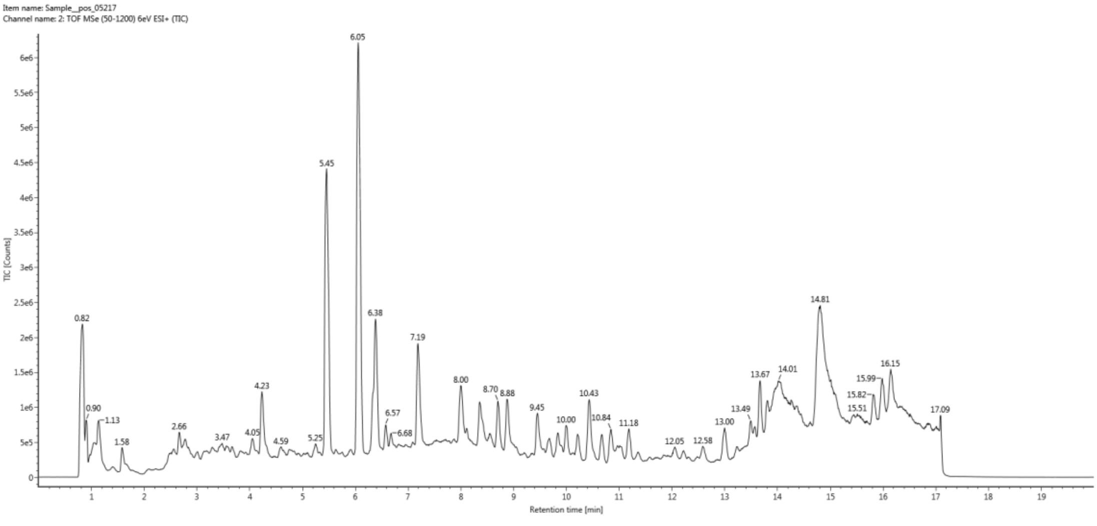
Item name: Sample__pos_05217
Channel name: 2: TOF MSe (50-1200) 6eV ESI+ (TIC)
TIC [Counts]
Retention time [min]

f

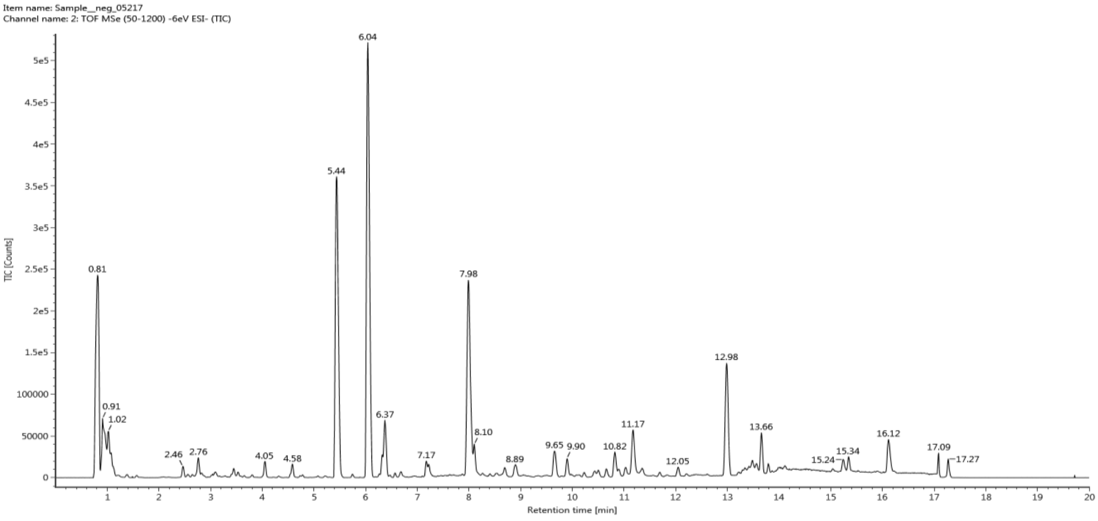
Item name: Sample__neg_05217
Channel name: 2: TOF MSe (50-1200) -6eV ESI- (TIC)
TIC [Counts]
Retention time [min]

g

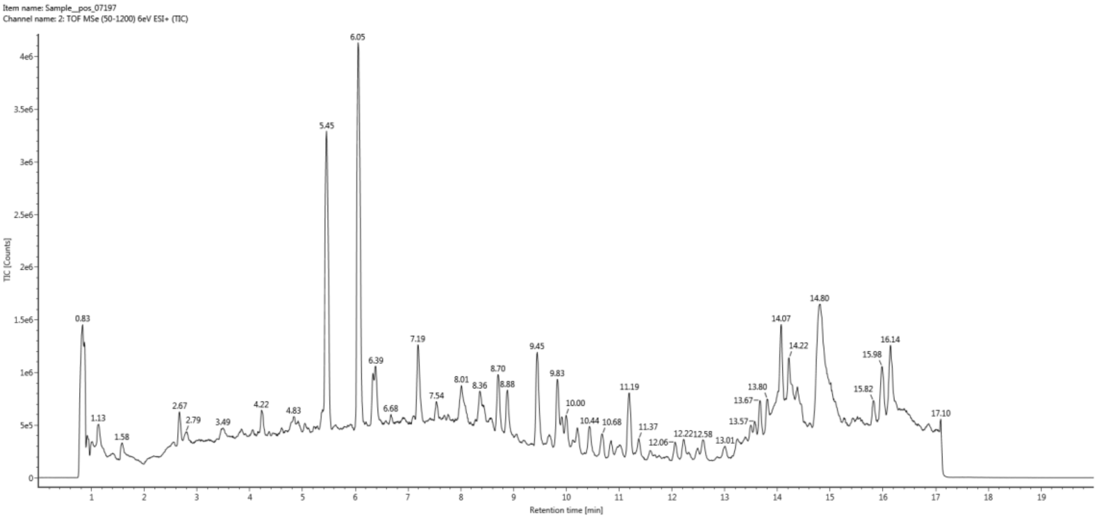

h

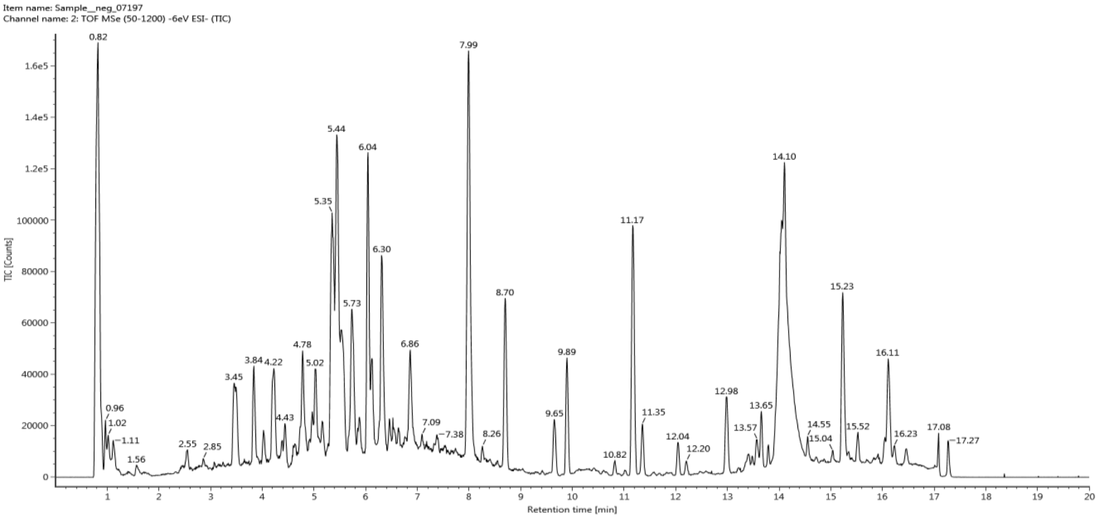

Figure S1 Total ion chromatogram (TIC) of germ substrate sample of *G. lucidum* by LC-QTOF-MS in positive ion mode and negative ion mode. a and c are TICs of L09-1 germ substrate at the beginning and the end of whole cultivation in positive ion mode; b and d are TICs of L09-1 germ substrate at the beginning and the end of whole cultivation in negative ion mode; e and g are TICs of L09-2 germ substrate at the beginning and the end of whole cultivation in positive ion mode; f and h are TICs of L09-2 germ substrate at the beginning and the end of whole cultivation in negative ion mode.

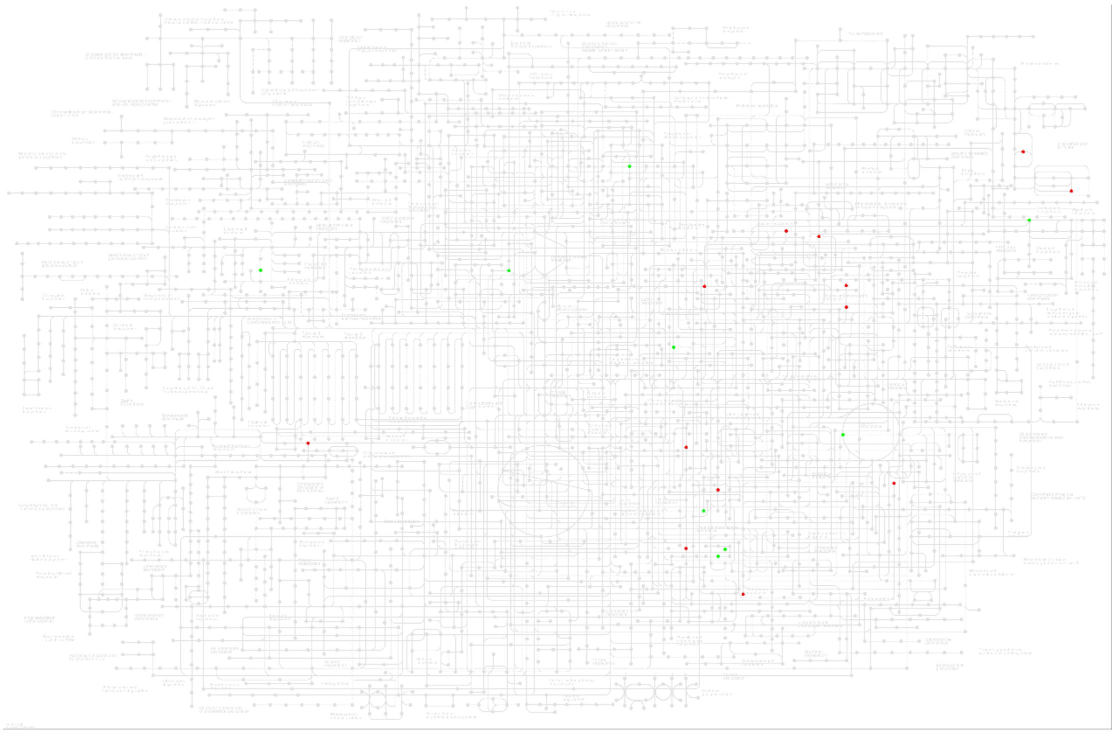

Figure S2 Metabolic pathway is the main pathway in two *G. lucidum* by KEGG pathway enrichment analysis. There were 22 acid compounds involved in positive ion mode and one acid in negative ion mode. The red points in metabolic pathways indicate that the acids were upregulated, and the green points perform the downregulated compounds.

a

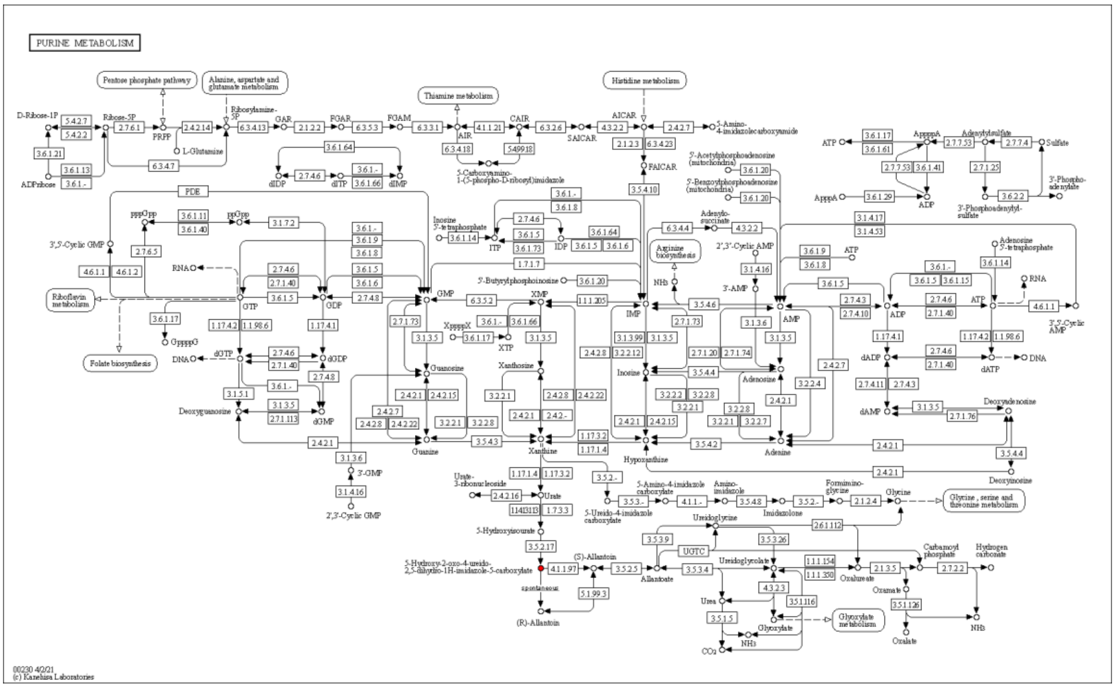

b

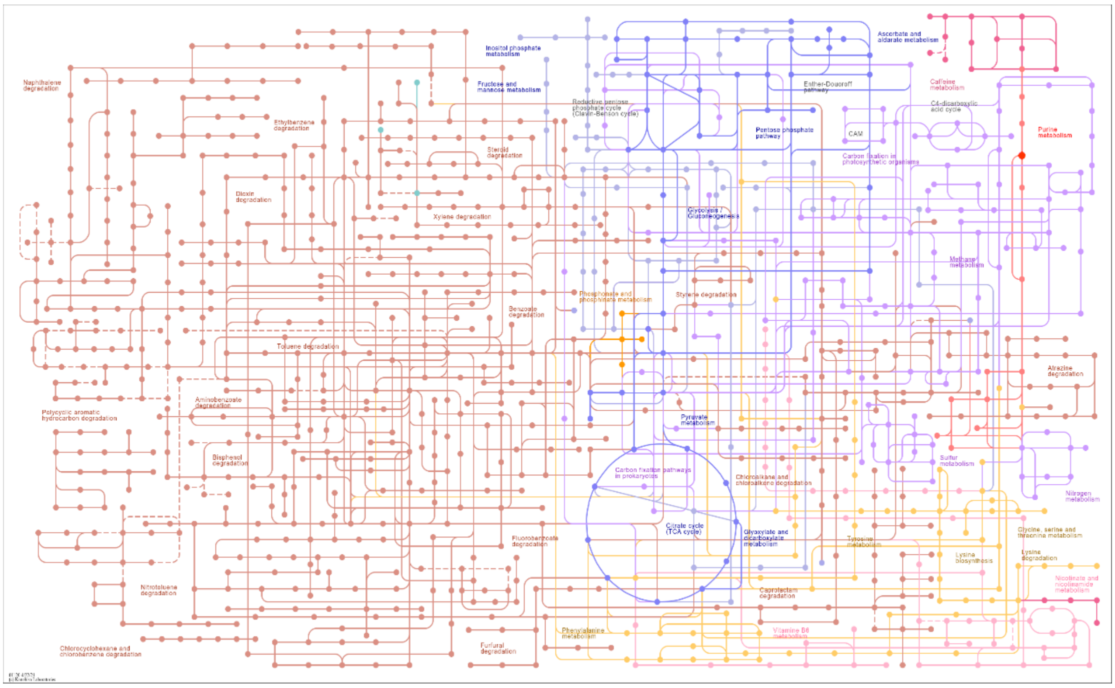

Fig.S3 5-hydroxy-2-oxo-4-ureido-2,5-dihydro-1H-imidazole-5-carboxylic acid, occurring in both modes and both cultivars, participates in purine metabolism (a) and microbial metabolism in diverse environment (b). The red points in pathways indicate the compound was upregulated significantly.

a

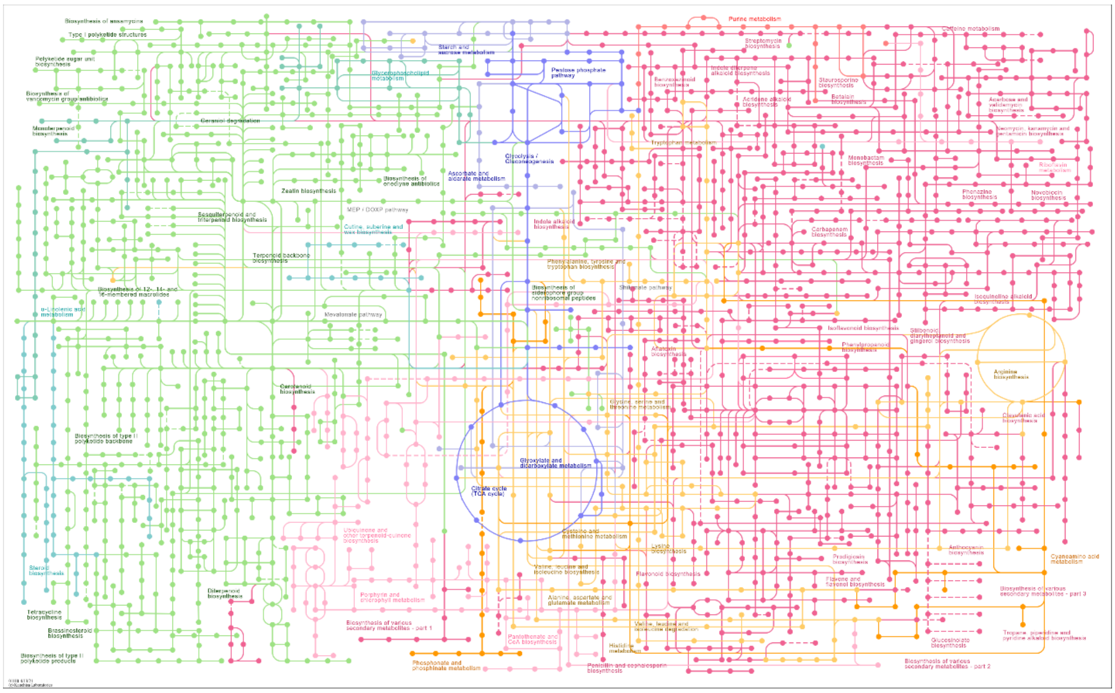

b

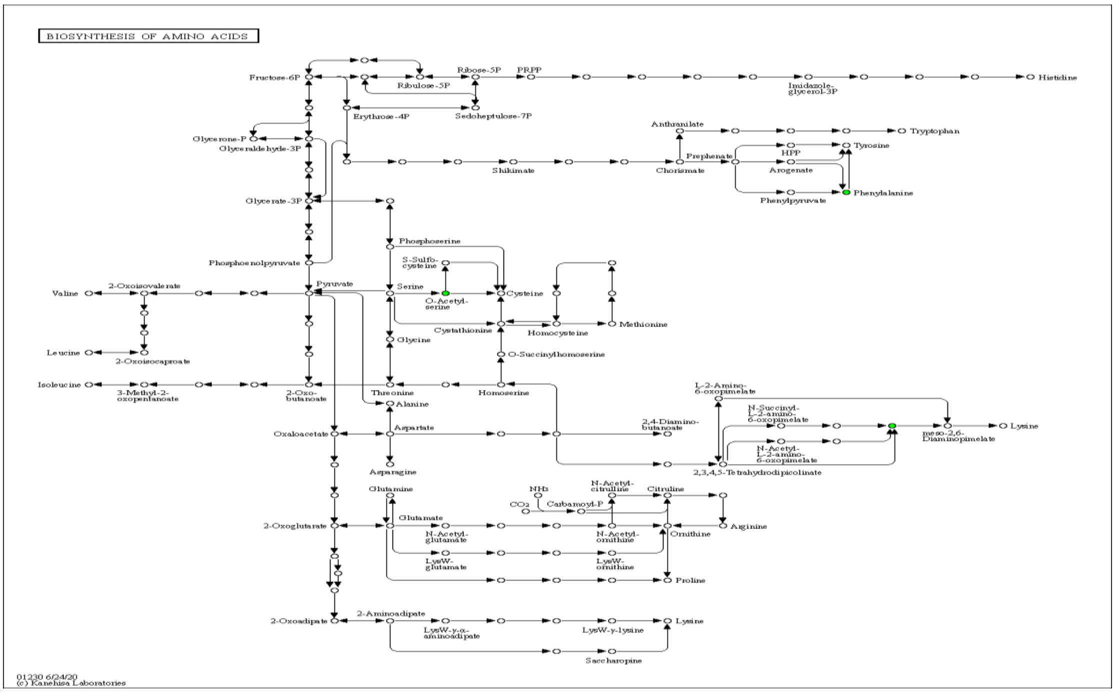
BIOSYNTHESIS OF AMINO ACIDS
Fructose-6P
Ribose-5P
PRPP
Ribulose-5P
Histidine
Imidazole-glycerol-3P
Erythrose-4P
Sedoheptulose-7P
Anthranilate
Tryptophan
Glycerone-P
Glyceraldehyde-3P
Prephenate
HPP
Tyrosine
Shikimate
Chorismate
Arogenate
Glycerate-3P
Phenylpyruvate
Phenylalanine
Phosphoserine
Phosphoenolpyruvate
S-Sulfo-cysteine
2-Oxoisovalerate
Pyruvate
Serine
Valine
Cysteine
O-Acetyl-serine
Methionine
Cystathionine
Homocysteine
Glycine
Leucine
2-Oxoisocaproate
O-Succinylhomoserine
Isoleucine
3-Methyl-2-oxopentanoate
2-Oxo-butanoate
Threonine
Homoserine
L-2-Amino-6-oxopimelate
Alanine
N-Succinyl-L-2-amino-6-oxopimelate
2,4-Diamino-butanoate
Aspartate
Oxaloacetate
meso-2,6-Diaminopimelate
Lysine
N-Acetyl-L-2-amino-6-oxopimelate
Asparagine
2,3,4,5-Tetrahydrodipicolinate
Glutamine
NH3
N-Acetyl-citrulline
Citruline
CO2
Carbamoyl-P
Glutamate
2-Oxoglutarate
Arginine
N-Acetyl-glutamate
N-Acetyl-ornithine
Ornithine
LysW-glutamate
LysW-ornithine
Proline
2-Aminoadipate
2-Oxoadipate
Lysine
LysW-γ-α-aminoadipate
LysW-γ-lysine
Saccharopine
01230 6/24/20
(c) Kanehisa Laboratories

c

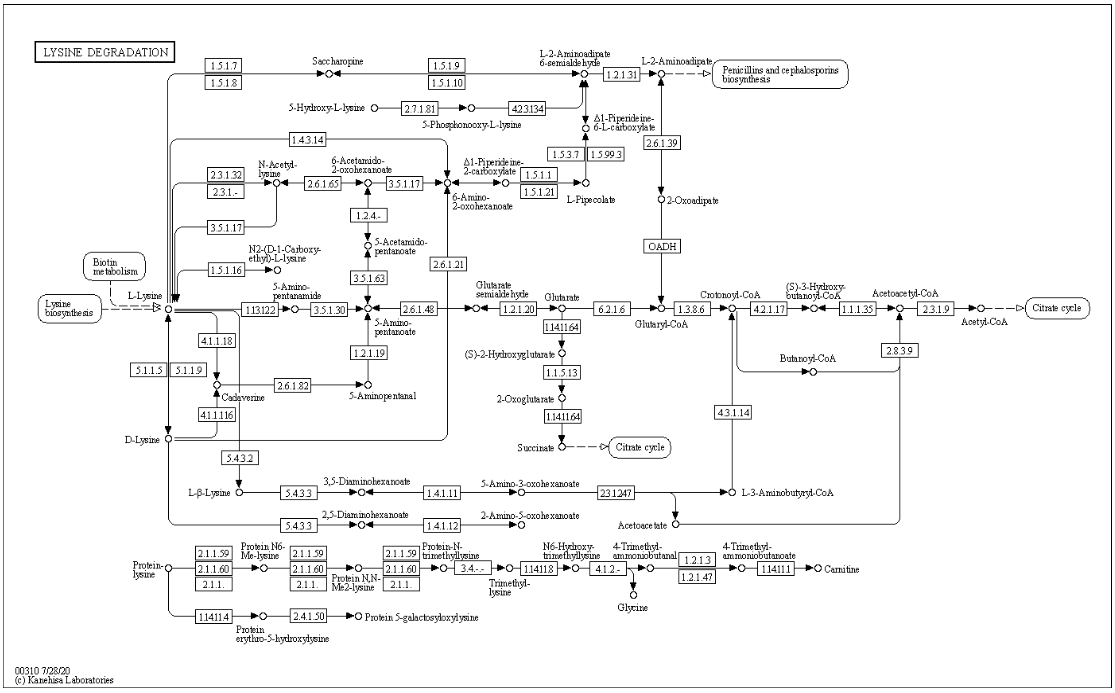

Figure S4 Related KEGG pathway of differential compounds in germ substrates of *G. lucidum*. a. 13 organic acids were involved in the biosynthesis of secondary metabolites. b. There were five organic acids participating in the biosynthesis of amino acids. c. There were three organic acids involved in Lysine degradation.
